# Q&A: How do plants respond to ethylene and what is its importance?

**DOI:** 10.1186/s12915-016-0230-0

**Published:** 2016-01-27

**Authors:** Caren Chang

**Affiliations:** Department of Cell Biology and Molecular Genetics, Bioscience Research Building, University of Maryland, College Park, Maryland 20742 USA

## Abstract

Ethylene gas is a major plant hormone that influences diverse processes in plant growth, development and stress responses throughout the plant life cycle. Responses to ethylene, such as fruit ripening, are significant to agriculture. The core molecular elements of the ethylene-signaling pathway have been uncovered, revealing a unique pathway that is negatively regulated. Practical applications of this knowledge can lead to substantial improvements in agriculture.

## What is the plant hormone ethylene?

The simple hydrocarbon ethylene (C_2_H_4_) is a tiny gaseous molecule of great significance. In addition to being the most widely produced organic compound in the world (used in manufacturing numerous products such as rubber, plastics, paints, detergents and toys), ethylene is a major hormone in plant biology. This volatile molecule mediates many complex aspects of plant growth, development and survival throughout the plant life cycle, including seed germination, root development, shoot and root growth, formation of adventitious roots, abscission of leaves and fruits, flowering, sex determination, and senescence of flowers and leaves [1, 2]. Ethylene also mediates adaptive responses to a variety of stresses, such as drought, flooding, pathogen attack and high salinity. During flooding, for instance, ethylene induces the formation of aerenchyma tissue (consisting of air-filled cavities) for oxygenation. Ethylene is best known, however, for its essential role in the ripening of climacteric fruits, such as tomatoes, bananas, pears and apples. Placing a ripe banana in a paper bag containing unripe avocados, for instance, will hasten ripening of the avocados due to the accumulation of ethylene produced by the banana.

## Why is ethylene important to agriculture?

Controlling ethylene responses is a major commercial enterprise due to the wide-ranging effects of ethylene on plants of agronomic and horticultural value [1]. Interestingly, responses to ethylene can be either harmful or desirable, depending on the species, developmental stage and concentration of ethylene. Too much ethylene, for example, can result in the spoilage of produce, as aptly conveyed by the saying “one bad apple spoils the whole bunch”. Costly methods are therefore employed to prevent the spoilage of fruits, vegetables and flowers during their transport and storage. These methods include the use of adsorbents and scrubbers to remove external ethylene, the use of chemical inhibitors to prevent ethylene biosynthesis and the use of chemical inhibitors (e.g., SmartFresh) to prevent ethylene signal transduction. Blocking ethylene perception during crop growth can also prevent abscission of leaves and flowers and yellowing of vegetables. On the other hand, ethylene is intentionally applied in situations where ethylene responses are desirable. Fruit ripening is typically induced pre- or post-harvest using ethylene or ethephon, which is a commercial liquid formulation of ethylene. Ethephon is also sprayed on pineapple plants to induce flowering and sprayed on wheat plants to prevent lodging (bending over).

## How was the ethylene hormone discovered?

Interestingly, the discovery of ethylene as a plant hormone came about due to the unintended presence of ethylene in the environment [1, 3]. In the 1800s, illuminating gas (coal gas) was widely used for lighting, and its leakage from gas lines was known to cause extensive damage to plants, such as the defoliation of trees around streetlamps. Near the end of the 1800s, Dimitry Neljubow observed that etiolated pea seedlings exhibited a peculiar growth (consisting of a shortened and thickened epicotyl and horizontal bending) due to leaking illuminating gas in his laboratory. Neljubow determined that ethylene was the biologically active component of illuminating gas. This finding led to numerous studies on the wide-ranging effects of ethylene. In 1934, Richard Gane discovered that plants synthesize ethylene; the correlation of ethylene biosynthesis with biological activity was a major step toward convincing researchers that a gas could be a plant hormone. In fact, ethylene was the first gaseous signaling molecule to be identified in any organism [4].

## Is there anything different about a gaseous hormone?

Ethylene is different from non-gaseous hormones in several ways. Ethylene moves within the plant by diffusion and is thought to be synthesized at or near its site of action, similar to the gaseous signal nitric oxide in mammals. Because ethylene can diffuse across membranes into nearby cells, there is no requirement for transporter proteins to deliver ethylene to target cells, and, in fact, no such transporters have been identified, though there is transport of the immediate precursor to ethylene, 1-aminocyclopropane-1-carboxylic acid (ACC) [5]. Ethylene is also not known to be conjugated or broken down for storage or deactivation; ethylene simply diffuses away from the plant. While managing a gaseous hormone is simpler for plants, it is more complicated for researchers. Ethylene experiments are generally carried out in contained environments, such as airtight chambers, although in some situations this can be circumvented by treating plants with ACC instead of ethylene.

## How do plants synthesize ethylene?

**Fig. 1:**
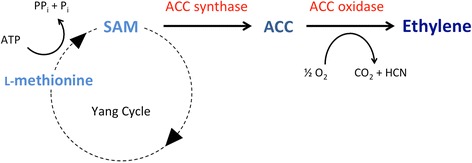
The two-step ethylene biosynthesis pathway in plants. In the first committed step, which is generally the rate-limiting step, ACC is synthesized from SAM by the enzyme ACS. SAM is produced from methionine in the “Yang cycle” of methionine cycling (named after Shang Fa Yang and colleagues, who elucidated the ethylene biosynthesis pathway in the 1970s). In the second step, ACC is converted to ethylene by the enzyme ACO

Plants synthesize ethylene using a two-step biochemical pathway starting from S-adenosyl-L-methionine (SAM) [5, 6] (Fig. 1). SAM is converted to ACC by the enzyme ACC synthase (ACS). ACC is then converted to ethylene by the enzyme ACC oxidase (ACO). The ACS and ACO enzymes are each encoded by a multigene family whose members are differentially expressed in response to internal developmental cues and environmental stresses, such as wounding, flooding, drought, mechanical pressure and pathogen attack [6]. Ethylene biosynthesis is also controlled by ACC synthase turnover, which is regulated by phosphorylation [6].

## How much ethylene is needed to trigger a response in plants?

Ethylene is biologically active at very low concentrations of around 0.01 to 1.0 part per million (ppm). Lower or higher sensitivities have been observed depending on the species and the response. Some climacteric fruits, such as tomatoes and apples, can generate tens of ppm of ethylene. It is worth noting here that ethylene is a byproduct of partial combustion of organic fuels and is present, therefore, in the atmosphere due to such things as forest fires, volcanic eruptions and car exhaust [1]. A study in 1973 detected up to 0.7 ppm of ethylene around the Beltway (the highway that circles Washington DC and the University of Maryland), and these levels had a harmful impact on the surrounding vegetation [7].

## Is ethylene harmful to humans?

Metazoans lack ethylene receptor homologs and do not perceive and respond to ethylene as plants do. High concentrations of ethylene (>1000 ppm) can, however, cause dizziness or light-headedness. For several decades in the 1900s, ethylene was used as a general anesthetic [8]. In ancient Greece, ethylene emanating from geologic faults beneath the Oracle of Delphi’s underground chamber may have been responsible for the oracles’ trance-like states and prophetic hallucinations [9]. The greatest danger in working with pure ethylene is the risk of explosion, because ethylene is a flammable gas. However, there is far too little ethylene in a tomato for it to explode!

## How was the ethylene-signaling pathway determined?

**Fig. 2:**
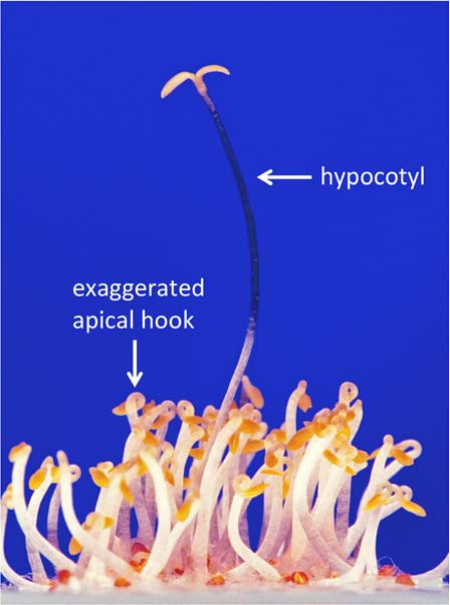
The triple response assay in *Arabidopsis*: a rapid method for screening for ethylene-response mutants. Dark-grown wild-type seedlings germinated on agar medium in the presence of exogenous ethylene display the triple response phenotype: a short and thick hypocotyl, an exaggerated apical hook (labeled in the figure) and a short root (not visible in this image). The single tall seedling (approximately 1 cm in length) is an ethylene-insensitive mutant with a long and thin hypocotyl (labeled in the figure) and no apical hook. From the cover of *Science* volume 241 (August 26, 1988); photograph by Kurt Stepnitz (Michigan State University). Reprinted with permission of AAAS

Major breakthroughs in understanding the ethylene-signaling pathway came from molecular genetic dissection of the pathway in the flowering plant *Arabidopsis thaliana*, initiated by the isolation of *Arabidopsis* ethylene response mutants. Mutants were isolated in the late 1980s concomitant with the development of *Arabidopsis* as a genetic plant model [10, 11], using a powerful genetic screen based on Neljubow’s observation in etiolated pea seedlings. In response to ethylene, etiolated *Arabidopsis* seedlings exhibit a short and thick hypocotyl, an exaggerated apical hook and a short root (Fig. 2). This phenotype, coined the “triple response”, is easily induced in the laboratory and is highly specific to ethylene. Cloning of the corresponding genes using map-based methods, such as chromosome walking, led to the identification of several key components of the pathway, including the first known plant hormone receptor, ETR1 [12]. Today, all of the central elements in ethylene signaling have been identified in *Arabidopsis*, and key mechanistic aspects of the pathway have been elucidated using a combination of genetics, molecular biology, cell biology and biochemistry*.* Studies in other plant species, particularly tomato [13], have further elaborated on and supported these findings. The ethylene-signaling pathway is highly conserved in plants (e.g., [13, 14]) and dates back to an algal ancestor prior to the colonization of land more than 450 million years ago [15].

## What does the ethylene-signaling pathway look like?

**Fig. 3:**
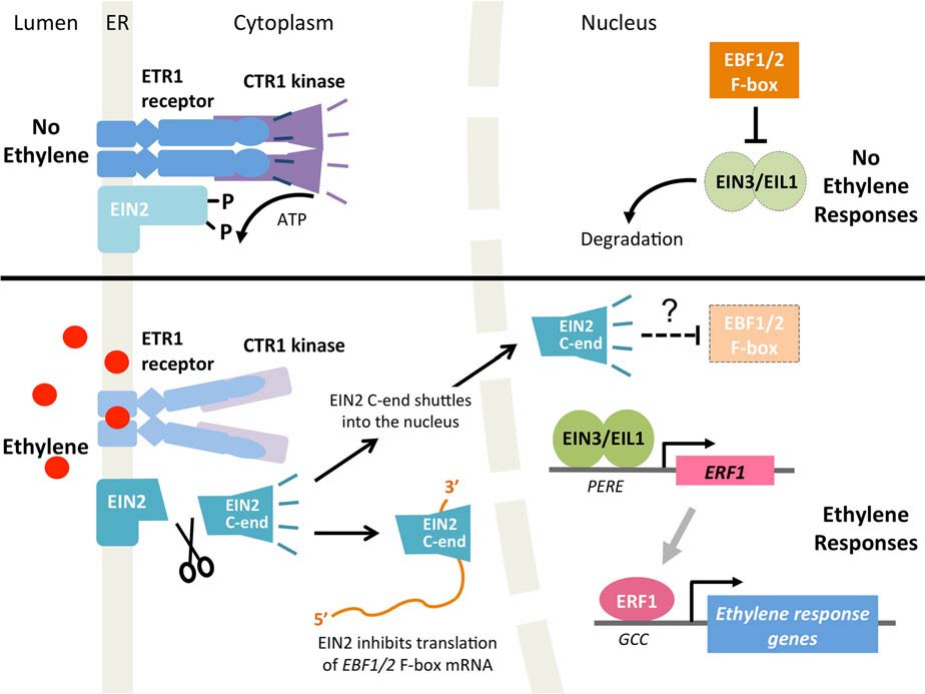
Model of the core ethylene-signaling pathway as described in the text. *Top*: in the absence of the ethylene signal, the ethylene receptors (represented by the isoform ETR1) activate the CTR1 protein kinase, which represses EIN2 function. In the nucleus, the master transcription factors EIN3/EIL1 are degraded. *Bottom*: when ethylene is detected, the ethylene receptors no longer activate CTR1, resulting in the proteolytic release of the EIN2 C-END, which inhibits protein translation of the F-box proteins EBF1/2. EIN3/EIL1 are consequently stabilized and regulate an extensive transcriptional cascade involving the ERF1 transcription factor. Other elements that regulate the pathway can be found in [37]

Ethylene signaling involves a unique pathway, shown in Fig. 3, that consists of the following main steps: (i) ethylene is perceived by an ethylene receptor complex at the endoplasmic reticulum (ER) membrane; (ii) ethylene detection triggers cleavage of a key protein in the complex, ETHYLENE-INSENSITIVE2 (EIN2); (iii) the cleaved soluble portion of EIN2 is involved in repressing the translation of two regulatory F-box proteins, which would otherwise target two master transcription factors for degradation by the 26S proteasome; and (iv) rapid stabilization of the two transcription factors results in the regulation of gene expression. The pathway relies heavily on negative regulation and post-translational controls. For example, as explained below, the ethylene receptors repress responses when no ethylene is detected (as opposed to activating responses when ethylene is detected), and the repression of ethylene responses involves protein phosphorylation and protein turnover (Fig. 3).

## What type of receptor is the ethylene receptor?

**Fig. 4:**
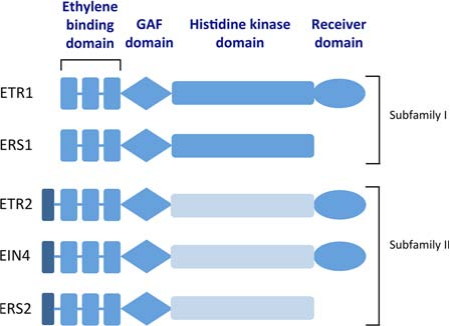
The five ethylene receptor isoforms in *Arabidopsis*. The ethylene-binding domain consists of three conserved transmembrane domains at the N-terminus (represented by the *vertical blue bars*). The receptors fall into two subfamilies. Subfamily II receptors have a fourth transmembrane domain (*dark blue*) at the N-terminus, which possibly serves as a signal sequence, and a degenerate histidine kinase domain (*light blue*) that displays serine/threonine kinase activity in vitro. Other plant species have similar ethylene receptor isoforms

The ethylene receptor is unexpectedly related to the histidine protein kinase receptors of the two-component signaling system, which is prevalent in prokaryotes but very rare in eukaryotes. It is believed that plants most likely acquired the ethylene receptor gene from an ancient endosymbiotic cyanobacterium that became the chloroplast [16]. Plants have a small family of ethylene receptors (e.g., *Arabidopsis* has five ethylene receptors and tomato has six) that have overlapping and distinct functions [16, 17]. As in typical prokaryotic two-component receptors, the ethylene receptor has an N-terminal ligand-binding domain followed by a GAF domain and a histidine protein kinase domain (Fig. 4). Some isoforms also have a C-terminal receiver domain, which is the second element of the two-component system (Fig. 4). In the ethylene receptors, the ethylene-binding domain lies within the ER membrane while the GAF, histidine kinase and receiver domains are in the cytoplasm. It is unclear why the ethylene receptors reside at the ER membrane, but given the diffusion of ethylene across membranes, there is no obligation for the receptor to be at the cell surface. Ethylene is more soluble in hydrophobic environments, consistent with the location of the ethylene-binding pocket within the membrane.

The ethylene receptors form disulfide-linked dimers, and each dimer is capable of binding a single ethylene molecule [18] with the help of a copper ion cofactor [19]. The dimers reside in clusters at the ER membrane where they interact with downstream proteins in the pathway [16, 17]. The GAF domain, usually known for binding small molecules, facilitates protein–protein interactions between ethylene receptor monomers as well as between isomers [16, 17].

## How do the ethylene receptors signal?

This is a good question. Currently this is best understood at the genetic level. From genetic analyses, we know that the receptors are negative regulators of ethylene responses. In other words, ethylene responses are repressed by ethylene receptor signaling [20]. This repression occurs in the absence of ethylene binding and is achieved through receptor activation of CONSTITUTIVE RESPONSE1 (CTR1), a serine/threonine protein kinase that has sequence similarity to the Raf protein kinase family [21]. CTR1 kinase activity negatively regulates the pathway (i.e., prevents downstream signaling) [21] (Fig. 3). When ethylene binds to the receptors, ethylene receptor signaling ceases. Consequently, CTR1 is no longer activated and downstream ethylene signaling can proceed (Fig. 3). This model is supported by the fact that null mutations in multiple ethylene receptor genes display constitutive ethylene responses similar to *ctr1* loss-of-function mutants, whereas dominant, gain-of-function receptor mutations confer ethylene insensitivity [20].

## What about the biochemical mechanism of ethylene receptor signaling?

This is still unresolved. In the canonical two-component system, binding of the ligand either stimulates or inhibits autophosphorylation of a conserved histidine residue followed by transfer of the phosphate to a conserved aspartate in the receiver domain. Curiously, histidine kinase activity does not appear to play a major role in ethylene receptor signaling [16, 17]. Although the ethylene receptors display histidine and/or serine/threonine kinase activity in vitro, neither activity has been definitively associated with ethylene signaling. In addition, despite hints of two-component signaling elements acting downstream of the receptors, there is strong evidence indicating that this is not the primary mode of ethylene signaling. Instead, the ethylene receptors physically associate with and signal to CTR1 [16]. The receptors also show interaction with the phosphorylation substrate of CTR1, ETHYLENE-INSENSITIVE2 (EIN2) [22].

Although genetic evidence indicates that ethylene binding inhibits receptor signaling, there is no clear answer to the basic question: Does the binding of ethylene stimulate or inhibit biochemical activity in the receptor? There are data to support each possibility. Although it might be counter-intuitive, a formal possibility is that CTR1 activation occurs by a passive (e.g., steric-based) signaling mechanism that is alleviated when ethylene receptor activity is triggered by the binding of ethylene. Complete structural data for the receptors, which is not yet available, will hopefully shed light on this question. It is also unclear why plants have multiple ethylene receptor isoforms. Although there is evidence that individual receptors have distinct roles in controlling specific responses, the underlying mechanisms for sub-functionalization are unknown [17].

## What happens downstream of the receptors in the ethylene signaling pathway?

Ethylene signaling downstream of CTR1 hinges on the phosphorylation status of EIN2, an enigmatic central regulator of the ethylene-signaling pathway [23]. EIN2 is tethered to the ER membrane by its N-terminal domain, which has sequence similarity to the widely conserved NRAMP metal ion transporters, but the biochemical function of this domain and its role in ethylene signaling have yet to be determined. The C-terminal portion (C-END) of EIN2 consists of a novel plant-specific domain that is cytosolic, and expression of this domain alone is sufficient for the activation of ethylene responses [23, 24]. In the absence of ethylene, the CTR1 kinase phosphorylates the EIN2 C-END, thereby preventing the C-END from signaling [25]. When the receptors detect ethylene, CTR1 is inactivated, and consequently the unphosphorylated EIN2 C-END is proteolytically released from the ER-anchored NRAMP domain [24, 25] (Fig. 3). The cleaved C-END then represses the translation of two F-box proteins, EIN3-BINDING F-BOX1 and 2 (EBF1/2), by binding to the 3’ untranslated regions of *EBF1/2* mRNA [26, 27]. This repression, which occurs within discrete cytoplasmic domains (known as P-bodies) where mRNA fates are decided, is crucial in ethylene signaling, because in the nucleus, the EBF1/2 proteins control the proteolytic degradation of two master transcription factors, EIN3/EIL1, which are required for essentially all known ethylene responses [28]. In the absence of ethylene, EBF1/2 target EIN3/EIL1 for ubiquitylation and degradation, in an SCF^EBF1/EBF2^ ubiquitin-ligating complex [28]; this is yet another example of negative regulation in the pathway. When ethylene is perceived, EIN2 represses translation of EBF1/2, thereby permitting the EIN3/EIL1 transcription factors to quickly accumulate in the nucleus, leading to rapid responses to ethylene. There is also evidence that the cleaved EIN2 C-END must enter the nucleus in order to activate downstream ethylene signaling, but the exact function of the C-END in the nucleus is unknown.

## What happens transcriptionally?

EIN3 was shown to initiate a transcriptional cascade that triggers several dynamic waves of gene expression that include feedback loops and the activation of genes known in numerous other hormone signaling pathways [29]. The primary targets of EIN3 include transcription factor genes in the *APETELA2* (*AP2*)*/ETHYLENE RESPONSE FACTOR* (*ERF*) family, such as *ERF1*, which regulates further expression in a transcriptional cascade of ethylene signaling [29, 30]. These global changes in gene expression result in a diverse array of cellular, metabolic and physiological responses [28].

## Where is ethylene research headed?

Given that plant growth, development and stress responses require the integration of diverse environmental and endogenous signals, there is a growing focus on ethylene cross-talk with other signals [31, 32]. Multiple points of ethylene cross-talk have been reported with the plant hormones auxin, gibberellins, brassinosteroids, abscisic acid, cytokinins and jasmonic acid. Cross-talk can occur through the regulation of ethylene biosynthesis, and more work is needed to elucidate such pathways. Conversely, ethylene signaling can induce the biosynthesis of other hormones. For instance, in deep water rice, ethylene signaling induces gibberellins, which signal internode elongation, allowing rice plants to escape from complete submergence [33]. Cross-talk can also occur in the ethylene-signaling pathway. For example, EIN3/EIL1 physically associate with transcription factors that are controlled by gibberellins [34] and jasmonic acid [35], promoting or inhibiting gene expression coordinately with these hormones. Given that *ein2* mutations have been obtained in genetic screens for components of other signaling pathways [23], EIN2’s role in translational regulation could be a point of signal integration as well.

Continued advances in understanding cross-talk and transcriptional networks will lead to a deeper understanding of ethylene-signaling networks that will someday allow for the modeling of specific plant responses. Applications of such knowledge have tremendous potential for agricultural improvements. Already, our understanding of ethylene biology can provide new strategies for manipulating ethylene responses, particularly by genetic means. Examples include the delay of flower senescence by expressing a dominant mutant *etr1-1* gene of *Arabidopsis* [36] and the breeding of flood-tolerant, high-yield rice plants by introducing an ethylene-inducible *ERF* transcription factor gene (*SUB1A*) [33]. The days of unknowingly manipulating ethylene biology, e.g., when ancient Chinese burned incense to ripen pears and early Egyptians gashed figs to induce their ripening [1], are far behind us.
